# Bioactive Films Containing Alginate-Pectin Composite Microbeads with *Lactococcus lactis* subsp. *lactis*: Physicochemical Characterization and Antilisterial Activity

**DOI:** 10.3390/ijms19020574

**Published:** 2018-02-14

**Authors:** Mariam Bekhit, Elmira Arab-Tehrany, Cyril J.F. Kahn, Franck Cleymand, Solenne Fleutot, Stephane Desobry, Laura Sánchez-González

**Affiliations:** 1 Laboratoire d’Ingénierie des Biomolécules (LIBio), ENSAIA-Université de Lorraine, 2 Avenue de la Forêt de Haye, TSA 40602, 54518 Vandœuvre-lès-Nancy CEDEX, France; mariamflear@yahoo.com (M.B.); elmira.arab-tehrany@univ-lorraine.fr (E.A.-T.); cyril.kahn@univ-lorraine.fr (C.J.F.K.); stephane.desobry@univ-lorraine.fr (S.D.); 2 Institut Jean Lamour (UMR CNRS 7198), Université de Lorraine, Parc de Saurupt, 54011 Nancy CEDEX, France; franck.cleymand@univ-lorraine.fr (F.C.); solenne.fleutot@univ-lorraine.fr (S.F.)

**Keywords:** biopolymer, hydrogel microbeads, corn starch, hydroxypropylmethylcellulose, mechanical properties

## Abstract

Novel bioactive films were developed from the incorporation of *Lactococcus lactis* into polysaccharide films. Two different biopolymers were tested: cellulose derivative (hydroxylpropylmethylcellulose (HPMC)) and corn starch. Lactic acid bacteria (LAB) free or previously encapsulated in alginate-pectin composite hydrogel microbeads were added directly to the film forming solution and films were obtained by casting. In order to study the impact of the incorporation of the protective culture into the biopolymer matrix, the water vapour permeability, oxygen permeability, optical and mechanical properties of the dry films were evaluated. Furthermore, the antimicrobial effect of bioactive films against *Listeria monocytogenes* was studied in synthetic medium. Results showed that the addition of LAB or alginate-pectin microbeads modified slightly films optical properties. In comparison with HPMC films, starch matrix proves to be more sensitive to the addition of bacterial cells or beads. Indeed, mechanical resistance of corn starch films was lower but barrier properties were improved, certainly related to the possible establishment of interactions between alginate-pectin beads and starch. HPMC and starch films containing encapsulated bioactive culture showed a complete inhibition of listerial growth during the first five days of storage at 5 °C and a reduction of 5 logs after 12 days.

## 1. Introduction

Lactic acid bacteria (LAB) were traditionally used for a technological purpose. Indeed, these bacteria provide taste, texture and increase the nutritional value of fermented foods such as dairy products (yoghurt, cheese), meat products, as well as some vegetables. However, a large amount of research has focused on a great potential of LAB use in food preservation. Studies have shown that LAB can inhibit the growth of different microorganisms, including bacteria, yeasts and fungi, through the production of organic acids, hydrogen peroxide, enzymes, defective phages, lytic agents and antimicrobial peptides, or bacteriocins [1].

During the last years, innovative bioactive films enriched with LAB have been developed [2,3,4]. Among biopolymers used as support for LAB, cellulose derivatives appear as remarkable film forming compounds. Not only are they biodegradable, odourless and tasteless [5] but they also exhibit good barrier properties against lipids, oxygen and carbon dioxide at low and intermediate relative humidity [6]. Hydroxypropylmethylcellulose has been used for instance for their good film forming properties and mechanical resistance. Another interesting polysaccharide used in active packaging is starch. This biopolymer is a renewable resource, inexpensive (compared with other compounds) and widely available [7].

However, one of the major problems encountered is the decrease of the film’s antimicrobial activity over time. Some studies connect this observation with problems of LAB viability [3,4]. To limit this problem and increase films effectiveness during time, encapsulation techniques appears as an interesting approach. Indeed, microencapsulation methods permit the entrapment of microbial cells within particles based on different materials and their protection against non-favorable external conditions [8]. Different factors such as encapsulation method, type and concentration of materials used, particle size and porosity or type of microparticles (bead, capsule, composite, coating layer) affect effectiveness of the bacterial protection [9]. Alginate has been widely used as microencapsulation material as it is non-toxic, biocompatible, and cheap [10,11,12]. Alginate consists in homopolymeric and heteropolymeric blocks alternating 1,4-linked β-d-mannuronic acid (M) and α-l-guluronic acid (G) residues in which the G units form crosslinks with divalent ions, to produce “egg-box” model gels. Ionic cross-linking gels are of great interest in drug release since destabilization and rupture of the gel can occur easily through replacement of multivalent cations with monovalent cations [13]. Studies have reported that alginate can form strong complexes with other natural polyelectrolytes such as pectin (also a polyuronate) by undergoing chain–chain association and forming hydrogels upon addition of divalent cations (e.g., Ca^2+^) [14,15], improving the mechanical and chemical stability of the alginate beads, and consequently improving the effectiveness of encapsulation [16].

The aim of the present study was to evaluate how the functionality of hydroxypropylmethylcellulose and corn starch films was affected by the incorporation of *L. lactis,* free or encapsulated in alginate-pectin composite microbeads, through the analysis of different physical properties (water vapor barrier, oxygen permeability, mechanical and optical properties) as well as their antilisterial effect.

## 2. Results and Discussion

### 2.1. Moisture Content and Thickness

Thickness average and moisture content in samples equilibrated at 5 °C and 75% relative humidity (RH) were reported in Table 1. The diameter of alginate/pectin microbeads was 274 ± 10 µm [17] and the thickness of pure HPMC films was 159 ± 6 µm. As expected, the addition of beads increases significantly HPMC films thickness because beads integrity was preserve in HPMC matrix. The thickness of HPMC films with microbeads was lower than beads diameter (210 ± 4 µm) certainly related to the dehydration of beads during film formation. In starch matrix no modifications were observed, this can be explained by the existence of possible interactions between alginate-pectin microbeads and starch. An increase of moisture content was equally reported in this case, which could be attributed to a greater water retention capacity. Lozano-Vazquez et al. [18] observed the IR spectra pattern of alginate beads changes with the addition of starch. The peak about 3390 cm^−1^ (wider for alginate beads containing starch) were attributed to starch-alginate interaction via the stretching of –OH groups.

### 2.2. Contact Angle Measurements

The surface wettability of the biopolymer films was measured by contact angle analysis using glycerol and diiodomethane. Results were reported on Table 2. No significant differences were observed among HPMC and corn starch matrices. The total energy values for HPMC and starch films were in the range of those reported by previous studies with biopolymer films [19,20]. The presence of LAB did not modify the wettability of films (data not presented in the table). However, concerning the addition of hydrogel microbeads results were different for HPMC and corn starch matrices. Indeed, contact angle was significantly modified following the addition of microbeads into starch matrix. This is certainly linked to the existence of interactions between alginate-pectin beads and starch as commented above.

We observed by adding the microbeads in the starch film the polar component increased significantly. This increasing presents the position of the microbeads at the surface of starch incompared to HPMC.

### 2.3. Barrier Properties

The water vapour permeability (WVP) and the oxygen permeability (OP) of the films at 0/75 RH gradient and 5 °C is also reported in Table 1. The WVP and OP of pure HPMC and corn starch films were in the range of those reported respectively by Klangmuang and Sothornvit [21] and Bonilla et al. [22]. Differences can be attributed to some modifications in the experimental conditions: RH gradient, temperature, kind and addition of plasticizer, etc. [23].

Under these experimental conditions, for both HPMC and corn starch films, the incorporation of lactic acid bacteria didn’t modify significantly WVP and OP values. Similar results were reported by Gialamas et al. [2]. The addition of bioactive culture didn’t affect significantly barrier properties of sodium caseinate films. Conversely, the use of microcapsules increases starch films barrier properties. This could be attributed to the possible establishment of interactions between alginate-pectin beads and starch already commented above in Section 2.1. This could limit the mass transfer of water molecules.

### 2.4. Mechanical Behaviour

Mechanical properties (in samples equilibrated at 75% and 5 °C) were measured in terms of the percentage of elongation at break (E%), tensile strength (TS) and elastic modulus (EM). TS represents the film’s resistance to elongation, E% is related with its stretching capacity and EM is a measure of the stiffness of the film. Results are reported in Table 1. Pure HPMC films were mechanically more stretchable (greater E% value) and less resistant to fracture (lower EM) than starch films.

When HPMC films contain alginate-pectin microbeads, only the percentage of elongation at break slightly changed (decreased). Hydrogel microbeads addition induces discontinuities and points of fragility within the polymeric matrix which implies a reduction of the cohesive forces in the polymer network. However, no significant changes were observed in terms of mechanical response after LAB addition for this type of films. Gialamas et al. [2] explained this little repercussion on the films mechanical properties by the relatively low mass of added bacterial cells.

The mechanical response of corn starch films was more affected by the presence of alginate-pectin microbeads and bacterial cells. After hydrogel microbeads incorporation, tensile strength at break and elastic modulus decreased. When *L. lactis* was incorporated in the matrix, an increase of percentage of elongation at break without notable changes tensile strength at break but a notable decrease of elastic modulus were observed. In comparison with HPMC films, starch matrix proves to be more sensitive to the addition of bacterial cells or alginate-pectin microbeads.

### 2.5. Optical Properties

Table 3 shows the optical properties of the films (color and transparency) since these properties have a direct impact on the appearance of the coated product.

Film transparency was evaluated through the internal transmittance at 450 nm, Ti (0–1, theoretical range). An increase in Ti can be assumed as an increase in transparency [24]. All films were highly transparent and no significant differences were observed depending on the nature of the polymer. Similar transparency values were reported in previous studies for HPMC [3] and starch [25]. The addition of alginate-pectin microbeads did not have a significant effect (*p* > 0.05) on the internal transmittance as compared with the control (pure HPMC or starch films). Conversely, the incorporation of lactic acid bacteria resulted in a slight decrease of the HPMC film transparency. This can be attributed to the presence of disperse bacterial cells, with a different refractive index, which enhance light scattering. It seems bacteria were better integrated in starch matrix.

As occurred with transparency, few changes are observed for the colour coordinates of the films following the addition of bacterial cells free or encapsulated (Table 2). The addition of microbial cells implied a decrease of HPMC films lightness and whiteness index.

Therefore, the addition of bioactive culture free or encapsulated in alginate-pectin beads didn’t modify the appearance of starch films but slightly affect optical properties of HPMC matrix. Indeed, these films become less transparent with lower lightness.

### 2.6. Antilisterial Activity

**Figure 1:**
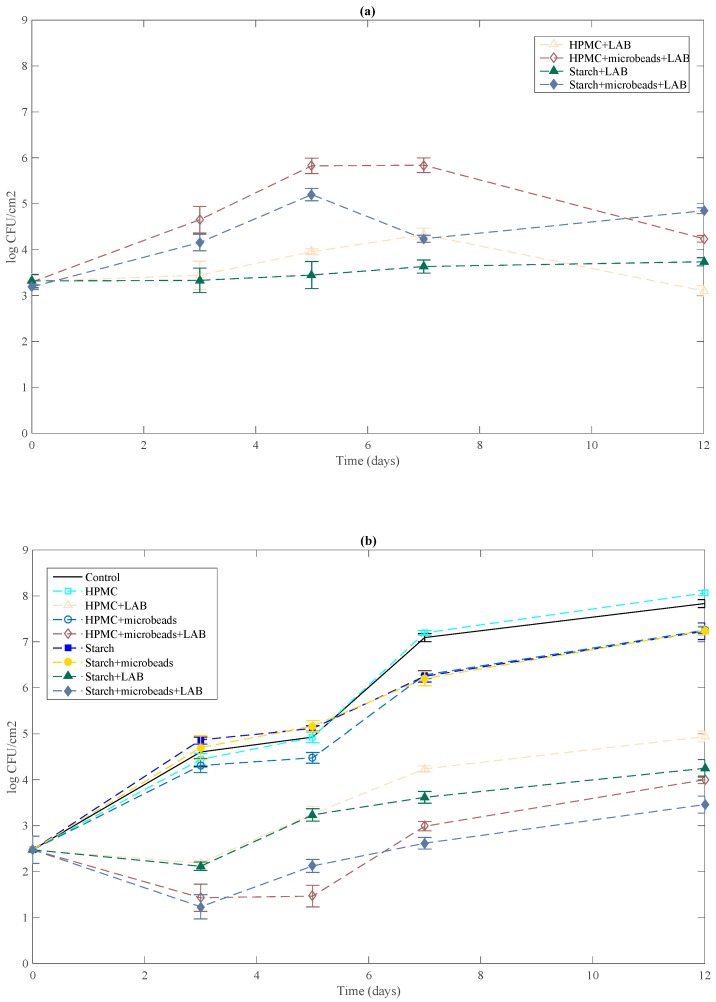
Effect of bioactive films on the growth of *L. monocytogenes* on TSA medium stored at 5 °C (**b**) and survival of LAB (*L. lactis*) in the film in contact with TSA (**a**). Mean values and standard deviation.

Counts of *L. lactis* are shown in Figure 1a. LAB encapsulated and added in HPMC or starch matrix grew immediately after the film came into contact with the surface of Tryptone Soy Agar (TSA), reaching a level in the order of 5.82 and 5.19 log CFU/cm^2^ respectively after 5 days at 5 °C and decrease to achieve an amount of 4.23 and 4.84 log CFU/cm^2^ at the end of the storage period. Bacterial survival in both matrices decreases significantly when *L. lactis* cells were incorporated directly. Differences of 1 log CFU/cm^2^ were observed between free cells and *L. lactis* encapsulated during all storage period in both matrices. Alginate-pectin beads protect effectively LAB.

The possible antilisterial effect at 5 °C of films was tested in a synthetic non-selective medium (TSA) medium and is shown in Figure 1b. Polysaccharide films without *L. lactis* were used as control as well as an uncoated plate. *L. monocytogenes* population increase from 2.47 to 7.83 log CFU/cm^2^ after 12 days of storage at 5 °C in uncoated TSA plates. As expected, polymeric matrices without lactic acid bacteria didn’t present antilisterial effect. No significant differences were observed between growth of *L. monocytogenes* on control TSA plates and plates coated with *L. lactis*-free films during storage period. Similarly, Sánchez-González et al. [3] found that *Listeria innocua* grew to more than 6 log CFU·cm^2^ after 10 days of storage at 5 °C with pure biopolymer films. Polysaccharide or protein (cellulose derivatives, sodium caseinate, pea protein) tested-based films were not effective by themselves to inhibit growth of pathogen bacteria. *L. lactis* incorporation into HPMC or starch matrices was effective to control *L. monocytogenes* growth. Following 3 days at 5 °C, bioactive films with free LAB reduced pathogen growth with respect to the control to 2.5 logs. The previous encapsulation of *L. lactis* into alginate-pectin beads improve results, since a reduction of 3 logs was achieved in this case after a 3 days’ storage period. Slight differences were observed respect to nature of biopolymer used. A significant antilisterial effect was still detected after 12 days of storage with bioactive films.

## 3. Materials and Methods

### 3.1. Materials

Sodium alginate from brown algae (viscosity ≤ 0.02 Pa·s for an aqueous solution of 1% wt at 20 °C), pectin from citrus peel (galacturonic acid ≥ 74%, Methoxy Groups ≥ 6.7%) and hydroxypropylmethylcellulose were purchased from Sigma-Aldrich (Sigma chemicals, St.-Louis, MO, USA). Corn starch was obtained from Roquette Laisa España (Benifaió, Spain) and calcium chloride dehydrate, sodium chloride, glycerol (99.5% AnalaR NORMAPUR) from WVR International (Darmstadt, Germany). Synthetic medium M17 and PALCAM agar were supplied by Biokar diagnostics (Beauvais, France), d(+)-Glucose monohydrate by Merck (Darmstadt, Germany).

Stock cultures of *L. lactis* ATCC 11454 and *L. monocytogenes* CIP 82110 were kept frozen (−80 °C) in synthetic media enriched with 30% glycerol (M17 Broth for LAB and Tryptone Soy Broth (TSB, Biokar diagnostics, Beauvais, France) for the other strain).

### 3.2. Microbeads Preparation

Alginate and pectin solutions (1% (*w*/*w*)) were prepared with sterile M17 broth supplemented with 0.5% d(+)-Glucose. Preliminary studies indicate a positive effect of addition of 0.5% d(+)-Glucose on *L. lactis* growth and nisin production. The polymers ratio (alginate/pectin) selected was 75/25. Bekhit et al. [17] reported that alginate/pectin (75/25) matrix with glucose-enriched M17 gave the best results when *L. lactis* was encapsulated. The physical properties and the entrapped efficiency of beads are greatly affected by the biopolymers ratio used. The best mechanical properties were found for alginate/pectin: 75/25; the beads were more stable and allow the best release of nisin during the storage period. The enrichment of internal medium with nutrients was key factor for bacteria viability and nisin production.

*L. lactis* culture was regenerated by transferring a loopful of the stock culture into 10 mL of M17 broth and incubated at 30 °C overnight. A 10 μL aliquot from overnight culture was again transferred into 10 mL of M17 broth and grown at 30 °C to exponential phase of growth (6 h). *L. lactis* cells were collected by centrifugation (20 min, 4 °C, 5000 rpm), washed twice with sterile sodium chloride solution (9%) and added to polymer mix. Bacterial suspension was correctly diluted to obtain a target inoculum in microbeads of 10^5^ CFU·mg^−1^.

Alginate-pectin hydrogel microspheres were made using the Encapsulator B-395 Pro (BÜCHI Labortechnik, Flawil, Switzerland). The Buchi technology is based on the principle that a laminar flowing liquid jet breaks up into equal sized droplets by a superimposed nozzle vibration. The vibration frequency determined the quantity of droplets produced and was adjusted at 1200 Hz to generate 1200 droplets per second. The flow rate was 3 mL·min^−1^. A 120 µm diameter nozzle was used for the preparation of beads. Droplets fell in a CaCl_2_ solution (100 mM) to allow microbeads formation. The beads were maintained in the gelling bath for 15 min to complete the reticulation process and then were filtered and washed with buffer solution (9% sodium chloride).

### 3.3. Preparation of the Bioactive Films

The film forming aqueous dispersions (FFD) contained 4% (*w*/*w*) of HPMC or corn starch and glycerol as plasticizer. The hydrocolloid:glycerol mass ratio was 1:0.25 in every case. Polymers were dissolved in distilled water (pH 6.5) under continuous stirring (400 rpm) at 25 °C.

*L. lactis* ATCC 11454 was used for the preparation of bioactive films. The selection of the strain was based on its antimicrobial activity, its ability to produce nisin, a bacteriocin. Microbial culture was regenerated according methodology described above. Lactic acid bacteria free or encapsulated were incorporated by adding the bacterial cells preparation into the FFD. The ratio was fixed in order to have a final concentration of 3 logs CFU/cm^2^ in dry film. FFD were then placed under magnetic stirring for 5 min.

A casting method was used to obtain the polysaccharide films without lactic acid bacteria and bioactive films. FFD were poured onto a framed and levelled Polyethylene Terephthalate (PET) Petri dishes (85 or 140 mm diameter) and were dried at 25 °C and 40% relative humidity for approximately 48 h. Film thickness was controlled by pouring the amount of FFD that will provide a surface density of solids in the dry films of 56 g/m^2^ in all cases. Dry films were peeled off the casting surface and preconditioned in desiccators at 5 °C and 75% relative humidity (RH) prior to testing. These values of temperature and RH were chosen to simulate the storage conditions of refrigerated coated products.

### 3.4. Films Characterization

#### 3.4.1. Moisture Content and Thickness

Moisture content was determined according the methodology described by Sánchez-González et al. [4]. After equilibration, films were dried in triplicate at 60 °C for 24 h in a natural convection oven and for 24 h more in a vacuum oven and the moisture content was calculated.

Measurements of film thickness were carried out by using an electronic digital micrometer (0–25 mm, 1 μm).

#### 3.4.2. Water Vapour Permeability

Water vapour permeability (WVP) was measured in dry film discs, which were equilibrated at 75% RH and 5 °C, according to the gravimetric method described in the AFNORN FH00-030 standard [26]. The dry film was sealed in a glass permeation cell containing silica gel, a dessicant. The glass permeation cells were 5.8 cm × 7.8 cm × 3.6 cm deep with an exposed area of 26.42 cm^2^. The permeation cells were placed in a controlled temperature (5 °C) and RH (75%) chamber via ventilation. The water vapour transport was determined from the weight gain of the cell. After 30 min, steady-state conditions were reached, and weightings were made. To calculate the water vapour transmission rate (WVTR), the slopes of weight gain as a function of time in the steady state period were determined by linear regression. For each type of film, WVP measurements were replicated three times and WVP was calculated according to Mc Hugh et al. [27].

#### 3.4.3. Oxygen Permeability

The oxygen permeability of the films (OP) was measured in triplicate by using an oxygen permeation measurement system (8100 Oxygen Permeation Analyser, Systech Illinois, UK) at 20 °C and 75% RH [28]. A sample of the film was placed in a test cell and pneumatically clamped in place. Films were exposed to pure nitrogen flow on one side and pure oxygen flow on the other side. An oxygen sensor read permeation through the barrier material and the rate of permeation or oxygen transmission rate was calculated taking into account the amount of oxygen and the area of the sample. Oxygen permeability was calculated by dividing the oxygen transmission rate by the difference in oxygen partial pressure between the two sides of the film, and multiplying by the average film thickness.

#### 3.4.4. Mechanical Properties

A Lloyd instruments universal testing machine (LRX-LLOYD, Lloyd Instruments, Fareham, Hants, UK) was used to determine the tensile strength (TS), elastic modulus (EM), and elongation (E) of the films, according to ASTM (American Society for Testing Materials) standard method D882 [29]. EM, TS and E were determined from the stress-Hencky strain curves, estimated from force-distance data obtained for the different films (2.5 cm wide and 10 cm long). At least six replicates were obtained for each formulation. Equilibrated film specimens were mounted in the film-extending grips of the testing machine and stretched at a deformation rate of 50 mm/min until breaking. The relative humidity of the environment was held constant at 53% during the tests, which were performed at 25 °C.

#### 3.4.5. Optical Properties

The transparency of the films was determined through the surface reflectance spectra in a spectrocolorimeter CM-5 (KonicaMinolta Co., Tokyo, Japan). Measurements were taken from three samples in each formulation by using both a white and a black background. The transparency was determined by applying the Kubelka-Munk theory for multiple scattering to the reflection spectra. As each light flux passes through the layer, it is affected by the absorption coefficient (K) and the scattering coefficient (S). Transparency was calculated, as indicated by Hutchings [24], from the reflectance of the sample layer on a white background of known reflectance and on an ideal black background, through the internal transmittance (Ti).

Colour coordinates of the films, L*, C_ab_* (Equation (1)) and h_ab_* (Equation (2)) from the CIELAB colour space were determined, using D65 illuminfant and 10° observer and taking into account *R*_∞_ (Equation (3)) which correspond with the reflectance of an infinitely thick layer of the material.

(1) $$C_{\mathrm{ab}}*=\sqrt{a{*}^{2}\;+\;b{*}^{2}}$$

(2) $$h_{\mathrm{ab}}*=arctg\left(\frac{b*}{a*}\right)$$

(3) $$R_{\infty }=a-b$$

Finally, the whiteness index (WI) was calculated by applying Equation (4).

(4) $$\mathrm{WI}=100-\sqrt{\left(100-L*\right)+a{*}^{2}\;+\;b{*}^{2}}$$

#### 3.4.6. Antimicrobial Activity of the Films against *L. monocytogenes*

Stock culture of *L. monocytogenes* CIP 82110 was regenerated by transferring a loopful into 10 mL of TSB and incubated at 37 °C overnight. A 10 μL aliquot from overnight culture was again transferred into 10 mL of TSB and grown at 37 °C to the end of the exponential phase of growth. Subsequently, this appropriately diluted culture was used for the inoculation of the agar plates in order to obtain a target inoculum of 10^2^ CFU/cm^2^.

The methodology followed for the determination of antimicrobial effectiveness of films was adapted from Kristo, et al. [30]. Aliquots of Tryptone Soy Agar (TSA, Biokar Diagnostics, Beauvais, France) (20 g) were poured into Petri dishes. After the culture medium solidified, properly diluted overnight culture from *L. monocytogenes* was inoculated on the surface and the different films (containing or not *L. lactis*) of the same diameter as the Petri dishes were placed onto the inoculated surfaces. Plates were then covered with parafilm to avoid dehydration and stored at 5 °C for 12 days. *L. monocytogenes* and *L. lactis* counts on TSA plates were examined both immediately after the inoculation and periodically during the storage period.

The agar was removed aseptically from Petri dishes and placed in a sterile plastic bag with 100 mL of tryptone soy water (Biokar Diagnostics, Beauvais, France). The bag was homogenized for 2 min in a Stomacher blender 400 (Interscience, Saint-Nom-La-Breteche, France). Serial dilutions were made and then poured onto M17 agar and PALCAM agar. Plates were incubated for 48 h at 37 °C before colonies were counted. All tests were run in duplicate.

#### 3.4.7. Surface Characterization

Static water contact angle (WCA) measurements of films before and after functionalization were performed using the sessile drop method with a contact angle instrument (Digidrop Contact Angle Meter, France) equipped with an image analysis attachment (Windrop, France). Uniform drops of liquids (0.75 μL) were carefully deposited on a horizontal film verso side (contact surface with Petri™ dish) using a micrometer syringe. The volume of the drops was kept constant since variations in the volume of the drops can lead to inconsistent contact angle measurements. Measurements were consistently conducted under the constant conditions of relative humidity (39%) and temperature (23 °C). Contact angle measurements were recorded three times on three different locations on the verso side within 5 s for a given blend thin film.

The total surface energy of the films was determined graphically by the Owens-Wendt method, which is usually applied for solids with low surface energy like polymers. The Owens-Wendt theory divides the surface energy into two components: one due to dispersive interactions and one due to polar interactions [31]. The total surface energy of a solid ($\gamma_{s}^{T}$) can be expressed as the sum of contributions from dispersive ($\gamma_{s}^{d}$) and polar (non-dispersive) ($\gamma_{s}^{p}$) force components. These can be determined from the contact angle, *θ*, of polar and non-polar liquids with known dispersive ($\gamma_{L}^{d}$) and polar ($\gamma_{L}^{p}$) parts of their surface energy, via the following equations:

(5) $$\gamma_{s}^{T}=\gamma_{s}^{d}+\gamma_{s}^{p}$$

(6) $$\gamma_{L\;}\left(1+\;\cos\theta \right)=2\sqrt{\gamma_{S}^{d}}\sqrt{\gamma_{L}^{d}}+\sqrt{\gamma_{S}^{P}}\sqrt{\gamma_{L}^{P}}$$

The total surface energy, polar component and dispersion component of films and functionalized films had been determined by the glycerol and diiodomethane contact angle using Digidrop contact angle meter apparatus.

### 3.5. Statistical Analysis

A statistical analysis of data was performed through a one-way analysis of variance using Statgraphics^®^ Plus for Windows 5.1 (Manugistics Corp., Rockville, MD, USA). Homogeneous sample groups were obtained by using Fisher’s least significant difference (LSD) test (95% significance level).

## 4. Conclusions

The present study revealed HPMC and corn starch matrices can act as effective carriers of *L. lactis* bacterial cells as antimicrobial agents and a previous encapsulation of *L. lactis* into alginate-pectin hydrogel beads improve the antilisterial effect. No significant differences were observed among nature of biopolymer film used, however microencapsulation of LAB increases cell viability during the 7 first days at 5 °C and consequently films antimicrobial effect. Indeed, HPMC and starch films containing bioactive encapsulated culture showed a complete inhibition of listerial growth during the first five days of storage at 5 °C and a reduction of 5 logs after 12 days.

The addition of bacterial cells, free or previously encapsulated, into the polymeric matrices did not alter significantly optical properties of the bioactive films. In comparison with HPMC films, starch matrix proves to be more sensitive to the addition of bacterial cells or alginate-pectin microbeads. Barrier properties were improved but mechanical resistance was lower.

Consequently, HPMC and corn starch films designed with alginate-pectin hydrogel microbeads as carrier of *L. lactis* bacterial cells appear as a promising biopreservation solution to limit listerial growth.

## Figures and Tables

**Table 1:** Effect of the incorporation of LAB (*L. lactis*) and microbeads on mechanical properties (elongation at break (E%), tensile strength (TS) and elastic modulus (EM)), water vapour permeability (WVP), oxygen permeability (OP), moisture content and thickness of biopolymer films equilibrated at 5 °C and 75% relative humidity. Mean values and standard deviation.

Film	E (%)	TS (MPa)	EM (MPa)	WVP (g·mm·kPa^−1^·d^−1^·m^−2^)	OP (cm^3^·m^−1^·Pa^−1^·s^−1^) × 10^7^	Moisture Content (g Water.g Film^−1^)	Thickness (µm)
HPMC	57 ± 7 ^a^	24 ± 4 ^a^	524 ± 45 ^a^	2.15 ± 0.11 ^a^	46 ± 3 ^a^	0.158 ± 0.002 ^a^	159 ± 6 ^a^
HPMC + microbeads	41 ± 7 ^b^	24 ± 3 ^a^	561 ± 26 ^a^	2.02 ± 0.11 ^a^	51 ± 8 ^a^	0.162 ± 0.002 ^a^	210 ± 4 ^b^
HPMC + LAB	58 ± 7 ^a^	25 ± 3 ^a^	473 ± 51 ^a^	2.25 ± 0.13 ^a^	43 ± 5 ^a^	0.153 ± 0.007 ^a^	153 ± 8 ^a^
HPMC + microbeads + LAB	34 ± 6 ^bd^	18 ± 4 ^ab^	447 ± 34 ^a^	2.22 ± 0.16 ^a^	55 ± 7 ^a^	0.154 ± 0.006 ^a^	205 ± 3 ^b^
Starch	3.3 ± 0.2 ^c^	20 ± 2 ^a^	962 ± 59 ^b^	3.05 ± 0.11 ^b^	2.50 ± 0.14 ^b^	0.166 ± 0.016 ^b^	123 ± 7 ^c^
Starch + microbeads	3.6 ± 0.3 ^c^	12 ± 4 ^b^	615 ± 100 ^c^	2.51 ± 0.11 ^c^	2.07 ± 0.06 ^c^	0.242 ± 0.006 ^c^	124 ± 4 ^c^
Starch + LAB	23 ± 4 ^d^	7.1 ± 0.5 ^c^	298 ± 58 ^d^	3.20 ± 0.11 ^b^	2.3 ± 0.2 ^b^	0.130 ± 0.002 ^d^	122 ± 4 ^c^
Starch + microbeads + LAB	31 ± 2 ^d^	6.0 ± 0.4 ^d^	280 ± 62 ^d^	2.42 ± 0.12 ^c^	1.91 ± 0.13 ^c^	0.273 ± 0.008 ^e^	125 ± 7 ^c^

^a, b, c, d, e^ Different letters in the same column indicate significant differences among formulations (*p* < 0.05).

**Table 2:** Contact angle and surface energy of biopolymer films equilibrated at 5 °C and 75% relative humidity. Mean values and standard deviation.

Film	Contact Angle (°)		Total Energy (mJ·m^−2^)	Polar Component (mJ·m^−2^)	Dispersive Component (mJ·m^−2^)
	Diiodomethane	Glycerol			
HPMC	55 ± 2 ^a^	83 ± 2 ^a^	31.3	0.8	30.4
HPMC + microbeads	55 ± 2 ^a^	87 ± 3 ^a^	32.4	0	32.4
Starch	51 ± 3 ^a^	83 ± 2 ^a^	33.9	0.07	33.8
Starch + microbeads	66 ± 3 ^b^	51 ± 2 ^b^	39.1	21.7	17.3

^a, b^ Different letters in the same column indicate significant differences among formulations (*p* < 0.05).

**Table 3:** Lightness (L*), chrome (C*_ab_), hue (h*_ab_), whiteness index (WI) and internal transmittance (Ti) of biopolymer films equilibrated at 5 °C and 75% relative humidity. Mean values and standard deviation.

Film	L*	(C*_ab_)	(h*_ab_)	WI	Ti (450 nm)
HPMC	81 ± 3 ^a^	1.8 ± 0.6 ^a^	104 ± 3 ^a^	73 ± 3 ^a^	86.2 ± 1.3 ^a^
HPMC + microbeads	79.1 ± 1.9 ^a^	2.3 ± 1.2 ^a^	99 ± 2 ^a^	72 ± 2 ^a^	85.7 ± 1.6 ^a^
HPMC + LAB	68.3 ± 0.6 ^b^	0.8 ± 0.4 ^a^	103.0 ± 1.5 ^a^	68.2 ± 0.6 ^b^	82.9 ± 1.4 ^b^
HPMC + microbeads + LAB	63 ± 2 ^c^	1.2 ± 0.2 ^a^	103 ± 2 ^a^	63 ± 2 ^c^	81.1 ± 1.4 ^b^
Starch	85.7 ± 1.3 ^d^	7.3 ± 0.7 ^b^	103.2 ± 1.8 ^a^	83.9 ± 0.9 ^d^	86.0 ± 1.2 ^a^
Starch + microbeads	73 ± 2 ^e^	6.8 ± 1.9 ^b^	102.4 ± 1.3 ^a^	80 ± 3 ^d^	83 ± 2 ^a^
Starch + LAB	86 ± 3 ^d^	5.0 ± 1.2 ^b^	99.3 ± 1.9 ^a^	86 ± 2 ^d^	87.5 ± 1.2 ^a^
Starch + microbeads + LAB	73 ± 4 ^e^	5.2 ± 1.7 ^b^	101.8 ± 1.2 ^a^	82 ± 2 ^d^	84.8 ± 1.6 ^a^

^a, b, c, d, e^ Different letters in the same column indicate significant differences among formulations (*p* < 0.05).

## Abbreviations

HPMC	Hydroxypropylmethylcellulose
LAB	Lactic Acid Bacteria
RH	Relative Humidity
WVP	Water Vapour Permeability
OP	Oxygen Permeability
E	Elongation
TS	Tensile Strength
EM	Elastic Modulus
Ti	Internal Transmittance
WI	Whiteness Index
TSA	Tryptone Soy Agar
L*	Lightness
C_ab_*	Chrome
h_ab_*	hue
TSB	Tryptone Soy Broth
FFD	Film Forming Aqueous Dispersions
PET	Polyethylene Terephthalate
WVTR	Water Vapour Transmission Rate
ASTM	American Society for Testing Materials
K	Absorption Coefficient
S	Scattering Coefficient
WCA	Water Contact Angle
LSD	Least Significant Difference
